# Early Emerging SARS-CoV-2 Spike Mutants Are Diversified in Virologic Properties but Elicit Compromised Antibody Responses

**DOI:** 10.3390/v15122401

**Published:** 2023-12-09

**Authors:** Junhao Fan, Shixiong Li, Yao Zhang, Jihao Zheng, Dongfang Wang, Yunxi Liao, Zhibo Cui, Dongyu Zhao, Dan H. Barouch, Jingyou Yu

**Affiliations:** 1 State Key Laboratory of Respiratory Disease, The First Affiliated Hospital of Guangzhou Medical University, Guangzhou 510182, China; fan_junhao@gzlab.ac.cn; 2 Guangzhou National Laboratory, Bio-Island, Guangzhou 510005, China; li_shixiong@gzlab.ac.cn (S.L.); zhang_yao2@gzlab.ac.cn (Y.Z.); zheng_jihao@gzlab.ac.cn (J.Z.); wang_dongfang@gzlab.ac.cn (D.W.); cui_zhibo@foxmail.com (Z.C.); 3 College of Life Sciences, Nankai University, Tianjin 300071, China; 4 Zhongshan School of Medicine, Sun Yat-sen University, Guangzhou 510080, China; 5 Department of Biomedical Informatics, School of Basic Medical Sciences, Peking University, Beijing 100191, China; 1911210039@pku.edu.cn (Y.L.); zhaodongyu@bjmu.edu.cn (D.Z.); 6 State Key Laboratory of Vascular Homeostasis and Remodeling, Peking University, Beijing 100191, China; 7 College of Pharmacy, Jinan University, Guangzhou 510632, China; 8 Center for Virology and Vaccine Research, Beth Israel Deaconess Medical Center, Harvard Medical School, Boston, MA 02215, USA

**Keywords:** SARS-CoV-2, early, spike, virology, immunology

## Abstract

Despite the effective antivirals and vaccines, COVID-19 remains a public health concern. The mutations that occurred during the early stage of the pandemic can be valuable in assessing the viral fitness and evolutionary trajectory. In this study, we analyzed a panel of 2969 spike sequences deposited in GISAID before April 2020 and characterized nine representative spike single-point mutants in detail. Compared with the WA01/2020, most (8 out of 9) mutants demonstrated an equivalent or diminished protein expression or processing, pseudovirus infectivity, and cell–cell fusion. Interestingly, most of the mutants in native form elicited minimum antibody responses in mice despite unaltered CD4+ and CD8+ T cell responses. The mutants remained sensitive to the antisera and the type I interferon. Taken together, these data suggest that the early emerging mutants are virologically divergent, and some of which showed transmission fitness. Our findings have important implications for the retrospective tracing of the early SARS-CoV-2 transmission and future pandemic preparedness.

## 1. Introduction

The coronavirus disease 2019 (COVID-19) has posed tremendous socioeconomic and scientific challenges to the global healthcare system and currently remains a threat as a result of the constant emergence of severe acute respiratory syndrome coronavirus 2 (SARS-CoV-2) mutations. As a single-stranded positive-sense RNA virus, SARS-CoV-2 tends to more rapidly accumulate more pronounced genomic mutations than its DNA counterparts, primarily ascribing to the intrinsically error-prone RNA-dependent RNA polymerase (RdRp) [1]. In addition, it has been reported that the host RNA-editing systems such as apolipoprotein B mRNA editing enzyme, catalytic polypeptide (APOBEC), and adenosine deaminase RNA-specific binding protein (ADAR) [2], as well as the recombination events involving co-infection of two viral lineages, may further fuel the diversification of a particular viral strain [3]. The enlarged mutation pool potentially creates new traits and offers selective advantages to the virus.

SARS-CoV-2 encodes four structural proteins, of which spike (S) serves as the main determinant that mediates viral entry and confers high immunogenicity. Spike proteins of coronaviruses are synthesized in the rough endoplasmic reticulum as a precursor SFL, where it is co-translationally glycosylated, trimerized, and subsequently transported to the Golgi apparatus where the proteolytic processing occurs [4]. In the trans-Golgi network, the SFL precursor is cleaved by cellular proteases such as furin into S1 and S2 subunits. The receptor binding domain (RBD) in the S1 subunit, as the name refers, is responsible for the engagement with the receptor angiotensin-converting enzyme 2 (ACE2), while the S2 subunit harbors the viral fusion machinery and, thus, governs the subsequent viral–host membrane fusion. Of note, the viral entry route of SARS-CoV-2 can be divergent, depending on the accessibility of co-factors for a second proteolytic cleavage [5]. The serine protease transmembrane protease, serine 2 (TMPRSS2), is required for SARS-CoV-2 entry via plasma membrane fusion, while endosomal cysteine proteases cathepsin B and L facilitate the viral entry through the endocytic pathway. The spike covers the external surface of the SARS-CoV-2 virion and, thus, the first and the most predominant encounter of the host immune cells. As a result, the generated immune responses concentrate on the spike protein. For instance, the protective neutralizing antibodies are virtually confined to the spike region, blocking the above-mentioned viral entry cascade. In addition, multiple dominant T cell epitopes have been identified in spike and suggested to confer protection against COVID-19 severe disease [6,7,8]. Therefore, the spike itself is central to the fulfillment of the viral life cycle and, most importantly, serves as the key immunogen that the host immune system targets. Consequently, the alterations in the spike domains may influence the virological features, including the capacity of the viruses to enter the cell, the transmissibility that the strains spread among the hosts, and the sensitivity to the prophylaxis and treatments; moreover, it could concomitantly lead to a change in the ability to provoke an immune response, occasionally ensuing an exacerbated viral pathogenicity.

The genetic alterations archived during the early or late phase of the pandemic can be different, depending on the selection pressure by natural immunity or vaccine-induced immunity. There is a plethora of evidence showing that a swarm of SARS-CoV-2 variants, in particular, the variants of concern (VOCs) that emerged after the massive vaccine deployment, showed enhanced immune evasion, accelerated transmissibility, and even aggravated pathogenicity [9,10,11,12]. The features of the early emerging mutants are less well-characterized, and their biological relevance remains largely elusive. The genotyping and phenotyping of the early mutants provide an efficient means to study the epidemiology of COVID-19 and develop diagnostic approaches. Moreover, it is crucial for assessing viral drug resistance and immune escape pathogenesis-related mechanisms. Computation simulations-based evolutionary and structural analysis predicted the features of early mutants, mainly in the RBD region. Hot spots such as V367F have been implicated by a few groups to possess a stronger binding affinity to the receptor ACE2 than the ancestral strain, while others, such as V483A, displayed diminished binding [13,14,15]. Interestingly, a few mutations, such as G476S, were predicted but with contradicting results. Regardless, these in silico models can be valuable, but a few traits need to be clarified, and more importantly, their biological relevance warrants further investigation.

In this study, we analyzed a panel of early SARS-CoV-2 spike mutants, characterized the virologic features, including protein expression, viral entry, and sensitivity to antivirals, and assessed their immunogenicity in eliciting antibody and T cell immune responses. The mixed traits provide us with an informative snapshot of the early phase of viral evolution, which may be conducive to COVID-19 origin tracing and future pandemic preparedness.

## 2. Materials and Methods

### 2.1. Plasmids, Cells, and Reagents

Sequences of a full-length spike (S) or 13 amino acids in cytoplasmic tail deleted S (SΔCT13) of SARS-CoV-2 (WA01/2020) were codon optimized and commercially synthesized (General Bio, Chuzhou, China). Synthetic genes were cloned into the mammalian expression plasmid pcDNA3.1+ (Invitrogen, Waltham, MA, USA). SARS-CoV-2 S D614G, V483A, S943P, D839Y, G476S, Q675H, V367F, W436R, N354D/D364Y variants were generated by site-directed mutagenesis based on SARS-CoV-2 SΔCT. Human ACE2 genes were commercially synthesized (General Bio, Chuzhou, China) and further cloned into a pQCXIP retroviral vector (Cat# 631516, Takara, San Jose, CA, USA). pHEF-VSVG expressing Vesicular Stomatitis Virus (VSV) glycoprotein was obtained through the NIH AIDS Reagent Program, Division of AIDS, NIAID, NIH (Cat# 4693). PsPAX2 (Cat#12260), pLenti-CMV-Puro-Luc (Cat#17447), and pBS-CMV-gag-pol (Cat#35614) were obtained from Addgene (Boston, MA, USA). IFNα2b (Cat# HY-P7023) was purchased from MedChemExpress, Monmouth Junction, NJ, USA.

HEK293T (ATCC^®^ CRL-11268™), Calu-3 (ATCC^®^ HTB-55™), TZM-bl (AIDS Reagent, Manassas, VA, USA, Cat# 8129) were maintained in Dulbecco’s modified Eagle’s medium (DMEM, Cat# C11995500BT, Gibco, Waltham, MA, USA), supplemented with 0.5% penicillin/streptomycin (Cat# 15070063, Gibco, Waltham, MA, USA) and 10% FBS (F8318, Sigma, St. Louis, MO, USA). HEK293T-hACE2 was maintained in DMEM, supplemented with 0.5% penicillin/streptomycin, 10% FBS, and 1 μg/mL puromycin (Cat# 540222, Sigma, St. Louis, MO, USA).

Mouse monoclonal antibody against SARS-CoV-2 RBD (Cat# 40592-MM117), rabbit polyclonal antibody against SARS-CoV-2 S2 (Cat# 40590-T62), and hACE2 conjugated with mouse IgG Fc (hACE2-mFc, Cat# 10108) was purchased from Sino Biological (Beijing, China). HRP-conjugated β-actin antibody (Cat# HRP-60008) was purchased from Proteintech (Rosemont, IL, USA). Rabbit polyclonal antibody against HIV1 p55 + p24 + p17 (Cat# ab63917) was purchased from Abcam (Cambridge, MA, USA).

Bafilomycin A1 (BafA1) was purchased from Cell Signaling Technology (Cas# 88899-55-2), E64d was kindly provided by Dr. Shaobo Wang (Guangzhou Laboratory, Guangzhou, China), while teicoplanin was provided by Dr. Xiancai Ma [16] (Guangzhou Laboratory, Guangzhou, China). A set of 15-mer peptides overlapping by 11 amino acids encompassing the spike (S) glycoprotein was synthesized by GL Biochem Ltd. (Shanghai, China) and used for the stimulation of T cells.

### 2.2. Selection of Early Emerging Spike Mutants

A total of 3165 whole genome sequences of SARS-CoV-2 sampled before April 2020 were downloaded from GISAID, and the workflow designed for SARS-CoV-2 was run using Nextstrain software, version 7.1.0 [17,18] (https://docs.nextstrain.org/projects/ncov/en/latest/ (accessed on 2 September 2023)). After multiple-sequence alignments were completed by Mafft [19] through the built-in program Augur, 2764 DNA sequences were aligned using WA01/2020 as the reference strain. Moreover, 2969 DNA sequences aligned to the spike gene, and the matching protein sequences were extracted from the intermediate file. The frequency of point mutations (non-synonymous mutations) was then measured by scanning each of the spike protein sequences’ amino acid positions one at a time. Nine spike mutations were filtered out for research based on the frequency of mutations after the N-terminal signal peptide area (the first 20 amino acids) and the C-terminal cytoplasmic tail region were excluded.

### 2.3. Generation of HEK293T-hACE2 Stable Cell

The retroviral pseudotypes expressing human ACE2 (hACE2) were generated by co-transfecting HEK293T cells with pHEF-VSVG, pBS-CMV-gag-pol, and pQCXIP-hACE2 at a ratio of 0.5:1:1. Viruses in the supernatants were collected every 12 h. The collected viruses were used to transduce HEK293T cells, and positive cell populations were selected by using 1 μg/mL puromycin.

### 2.4. Production of Pseudotyped Lentiviral Particles

The SARS-CoV-2 pseudoviruses expressing a luciferase reporter gene were generated in an approach similar to as described previously [20,21]. Moreover, 10 µg packaging construct psPAX2, 10 µg luciferase reporter plasmid pLenti-CMV Puro-Luc, and 5 µg spike protein expressing pcDNA3.1-SARS-CoV-2 SΔCT were co-transfected into 5 × 10^6^ HEK293T cells in T-75 flask with lipofectamine 2000 (Sigma, St. Louis, MO, USA). Six hours post-transfection, the supernatants were replaced with fresh DMEM (plus 5% FBS). The supernatants containing the pseudotype viruses were collected 48 h post-transfection; pseudotype viruses were purified by filtration with a 0.45 µm filter.

### 2.5. Cell–Cell Fusion Assay

HEK293T cells were transfected with spike-encoding plasmids, along with HIV-1 Tat optimized expression vector (AIDS Reagent, Manassas, VA, USA, Cat# 827). Meanwhile, TZM-bl cells were transfected with human ACE2 plasmid. Twenty-four hours after transfection, cells were cocultured with TZM-bl at a 1:1 ratio for 12 h, lysed, and measured for firefly luciferase activity. All samples were tested in duplicates, and the results were averaged.

### 2.6. Virus Entry and Kinetics

In a flat 96-well plate, 1.5 × 10^4^ HEK293T-ACE2 cells/well were seeded at a density of 1.5 × 10^5^ cells per mL. On the second day, 50 µL of equal amount of p24 virus (approximately 20 ng) was added into each well and spun at 1680 g, 30 min, 4 °C. The cell culture plates were transferred into a 37 °C incubator immediately after spin to initiate viral internalization and infection. Moreover, 15 µL DMEM medium containing 50 nM BafA1 was added into each well immediately after spinoculation (0 h) and 10 min, 1, 2, 3, 4, 6, and 8 h post-temperature shift. Furthermore, 12 h after culturing, cells were replenished with fresh DMEM. Additionally, 48 h post spinoculation, cells were lysed for luciferase activity quantification.

### 2.7. Western Blot

Western blot was performed as previously described [22]. Briefly, transfected HEK293T cells were collected, washed once with icy 1 × PBS (Cat# BL302A, Biosharp, Hefei, China), and lysed in Radioimmunoprecipitation assay buffer (RIPA, Cat# P0013B, Beyotime, Nantong, China) for 20 min on ice. The pseudoviral particles were obtained from DMEM culture medium by centrifugation (4000 rpm, 10 min, 4 °C). The cell lysates and viral particles were dissolved in sample buffer (Cat# P1041, Solarbio, Beijing, China), separated on 20% gradient gel (Cat# M00657, GenScript, Benelux, The Netherlands), and detected by anti-HIV-1 p24, anti-SARS-CoV-2 RBD, anti-SARS-CoV-2 S2, and anti-β-actin antibodies.

### 2.8. Cell Surface Staining

Cells were washed twice with cold 1× DPBS plus 2% FBS, detached with 1× DPBS containing 5 mM EDTA, and incubated on ice with the appropriate primary antibodies for 1 h. After three washes with PBS plus 2% FBS, cells were further incubated with FITC-conjugated secondary antibodies for 45 min. After two washes, cells were fixed with 2% formaldehyde and analyzed in a BD LSRII flow cytometer.

### 2.9. Animals and Study Design

Additionally, 75 specific pathogen-free 6- to 8-week-old BALB/c mice were purchased from Hunan SJA Laboratory Animal Co., Hunan, China, and maintained in the Animal Care Facilities at the Guangzhou Laboratory and were randomly allocated to 12 groups. Mice received pcDNA3.1 vectors expressing different SARS-CoV-2 S protein or sham controls (*n* = 5 or 10 per group). Animals received a single immunization of 50 μg of plasmid by the intramuscular route without adjuvant. Then, 4 weeks after prime, the mice were boosted with another shot of 50 μg of plasmid. At indicated time points, peripheral blood was collected via the submandibular route to isolate serum for immunologic assays.

To test sensitivity of early spike mutants to antiserum, eight ICR mice (Gempharmatech Co., Ltd., Nanjing, China) were intramuscularly immunized with 0.5 µg (*n* = 2), 5 µg (*n* = 3), or 50 µg (*n* = 3) of spike ferritin nanoparticle vaccine adjuvanted with 100 µg of Alhydrogel (Invivogen, San Diego, CA, USA) at weeks 0 and 4. At week 6, peripheral blood was collected via the submandibular route to isolate serum for immunologic assays.

All animal studies were conducted in compliance with all relevant local and national regulations and were approved by the Institutional Animal Care and Use Committees of Guangzhou Laboratory.

### 2.10. Lentiviral Luciferase-Based Neutralization Assay

The SARS-CoV-2 pseudoviruses neutralization assay was generated in an approach similar to that described previously [20,21]. To determine the neutralization activity of the serum, plasma, or IgG samples from cohorts, HEK293T-hACE2 cells were seeded in 96-well tissue culture plates at a density of 1.75 × 10^4^ cells/well overnight. Three-fold serial dilutions of heat-inactivated plasma samples were prepared and mixed with 50 µL of pseudovirus. The mixture was incubated at 37 °C for 1 h before being added to HEK293T-hACE2 cells. Moreover, 48 h after infection, cells were lysed in Steady-Glo Luciferase Assay (Promega) according to the manufacturer’s instructions. SARS-CoV-2 neutralization titers were defined as the sample dilution at which a 50% reduction in the relative light unit (RLU) was observed relative to the average of the virus control wells.

### 2.11. Enzyme-Linked Immunosorbent Assay (ELISA)

SARS-CoV-2 receptor-binding domain (RBD)-specific binding antibodies in serum were assessed by ELISA. Additionally, 96-well plates were coated with 1 μg/mL of similarly produced SARS-CoV-2 WA1/2020, RBD protein in 1× Dulbecco phosphate-buffered saline (DPBS) and incubated at 4 °C overnight. After incubation, plates were washed once with wash buffer (0.05% Tween 20 in 1× DPBS) and blocked with 350 μL of casein block solution per well for 2 to 3 h at room temperature. Following incubation, the block solution was discarded, and the plates were blotted dry. Serial dilutions of heat-inactivated serum diluted in casein block were added to wells, and plates were incubated for 1 h at room temperature prior to 3 more washes and a 1 h incubation with a 1 μg/mL dilution of anti-mouse IgG horseradish peroxidase (HRP) (Invitrogen, ThermoFisher Scientific, Waltham, MA, USA) at room temperature in the dark. Plates were washed 3 times, and 100 μL of SeraCare KPL TMB SureBlue Start solution was added to each well; plate development was halted by adding 100 μL of SeraCare KPL TMB Stop solution per well. The absorbance at 450 nm was recorded with a VersaMax microplate reader (Molecular Devices, San Jose, CA, USA). For each sample, the ELISA end-point titer was calculated using a 4-parameter logistic curve fit to calculate the reciprocal serum dilution that yields an absorbance value of 0.2 at 450 nm. Interpolated end-point titers were reported.

### 2.12. Intracellular Cytokine Staining (ICS) Assay

CD4+ and CD8+ T cell responses were quantitated by pooled peptide-stimulated intracellular cytokine staining (ICS) assays. Peptide pools were 15 amino acid peptides overlapping by 11 amino acids spanning the SARS-CoV-2 WA1/2020. Moreover, 2 × 10^5^ peripheral blood mononuclear cells well were re-suspended in 100 µL of R10 media supplemented with CD49d monoclonal antibody (1 µg/mL) and CD28 monoclonal antibody (1 µg/mL). Each sample was assessed with mock (100 µL of R10 plus 0.5% DMSO; background control), peptides (2 µg/mL), and/or 10 ng/mL phorbol myristate acetate (PMA) and 1 µg/mL ionomycin (Sigma-Aldrich, St. Louis, MO, USA) (100 µL; positive control) and incubated at 37 °C for 1 h. After incubation, 0.25 µL of GolgiStop and 0.25 µL of GolgiPlug in 50 µL of R10 was added to each well and incubated at 37 °C for 8 h and then held at 4 °C overnight. The next day, the cells were washed twice with DPBS, stained with aqua live/dead dye for 10 min and then stained with predetermined titers of monoclonal against CD8a (clone 53–6.7; BUV395), CD3 (clone 17A2; BUV737), CD103 (clone 2E7; BV605), CD44 (clone IM7; BV711), CD62L (clone MEL-14; Alexa Fluor 488), CD4 (clone RM4–5; PE/Dazzle 594), and CD69 (clone H1.2F3; PE-Cy7) (all antibodies diluted 1:100) for 1 h. Cells were then washed twice with MACS buffer and incubated with 100 µL of BD Cytofix/CytoPerm Fixation/Permeabilization solution for 15 min. Cells were then washed twice with 1x Perm Wash buffer (BD Perm/WashTM Buffer 10× in CytoFix/CytoPerm Fixation/Permeabilization kit diluted with MilliQ water and passed through 0.22 µm filter) and stained intracellularly with mAbs against IFN-γ (clone XMG1.2; BV421), IL-2 (clone JES6-5H4; PE), IL-4 (clone 11B11; APC), and TNFα (clone MP6-XT22; Alexa Fluor 700) (all antibodies diluted 1:100) for 1 h. Cells were then washed twice with 1× Perm Wash buffer and washed once with MACS buffer, then fixed with 250 µL of freshly prepared 2% formaldehyde. Fixed cells were analyzed by the BD LSRII system. Data were analyzed using FlowJo v9.9.

### 2.13. ELISPOT

ELISPOT plates were coated with rat anti-mouse IFN-γ monoclonal antibody (BD Biosciences, Franklin Lakes, NJ, USA, 554410) diluted 1:100 at a final concentration of 500 ng per well overnight at 4 °C. Plates were washed with DPBS containing 0.25% Tween 20, and blocked with R10 media (RPMI with 10% FBS and 1% penicillin–streptomycin) for 1 h at 37 °C. The Spike peptide pools contain 15 amino acid peptides overlapping by 11 amino acids that span the protein sequence. Spike was prepared at a concentration of 2 µg per well, with 500,000 cells per well added. The peptides and cells were incubated for 18–24 h at 37 °C. All steps following this incubation were performed at room temperature. The plates were washed with coulter buffer and incubated for 2 h with Rat anti-mouse IFN-γ Biotin (diluted 1:200) from BD Biosciences, Franklin Lakes, NJ, USA (375 ng/well). Plates were washed again and then incubated with Streptavidin–alkaline phosphatase antibody from Southern Biotechnology, Birmingham, AL, USA (7105–04) (20 µL/well). The third wash was followed by the addition of Nitor-blue Tetrazolium Chloride/5-bromo-4-chloro 3′ indolyl phosphate p-toluidine salt (NBT/BCIP chromagen) from Thermo Fisher Scientific, Waltham, MA, USA for 7 min. Chromagen was discarded, and plates were washed with water and dried in a dark place for 24 h. Plates were scanned and counted on a Cellular Technologies Limited Immunospot Analyzer.

### 2.14. Statistical Analyses

Descriptive statistics and logistic regression were performed using GraphPad Prism 9.0.0 (GraphPad Software, San Diego, CA, USA). Immunologic data were generated in duplicate and were compared by two-sided Mann–Whitney tests. Correlations were assessed by two-sided Spearman rank-correlation tests. *p* values less than 0.05 were considered significant.

## 3. Results

### 3.1. Early Emerging Mutations Dispersed the Entire Spike Protein

**Figure 1:**
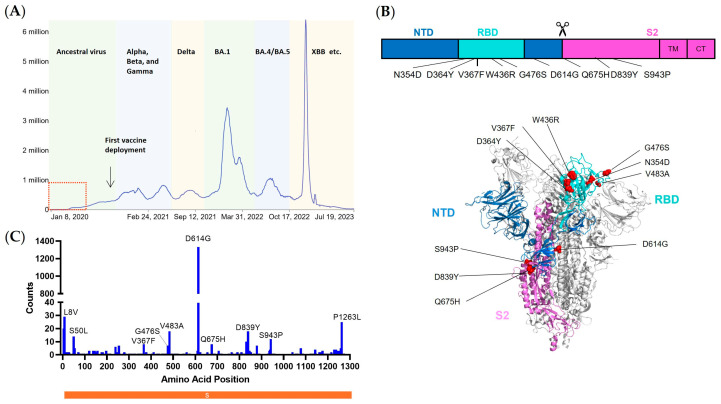
Early emerging mutations dispersed the entire spike protein. (**A**) Schematic demonstration of daily new confirmed COVID-19 cases with a 7-day rolling average. Data are derived from the WHO COVID-19 dashboard. The first vaccine deployment was initiated in December 2020, and the dashed square indicates the time period of sampling. (**B**) Distributions of mutations in the spike region and the ones with relatively high frequencies were labeled. (**C**) Schematic illustration of WA01/2020 S primary structure colored by domain (upper) and the structure of the closed prefusion conformation of the WA01/2020 S trimer (lower) is shown in ribbon diagram with one protomer colored as NTD in blue, RBD in cyan, and S2 in purple. All mutations in the early mutants, as compared with the WA01/2020 spike, are highlighted in the sphere model. Models were generated using the structure of SARS-CoV-2 (Protein Data Bank code 7DK3).

To specifically characterize the features of mutations accumulated during the early phase of the COVID-19 pandemic without the interference of the anti-SARS-CoV-2 specific immunity, we designed to select the SARS-CoV-2 whole genome sequences collected and deposited in GISAID before April 2020, a time when the first vaccine deployment was not initiated and the reinfections were unlikely to happen (Figure 1A). A total of 2969 neat sequences of spike were obtained after excluding ones with incomplete or blurring nucleotides, with either base-calling errors, unsolved “Ns”, or undefinable gaps. Annotated spike protein sequences were aligned, and the frequency of individual point mutations was identified in comparison with the ancestral strain WA01/2020 (Figure 1B). Interestingly, the non-synonymous mutations dispersed throughout the entire spike genome positions (Figure 1B,C). A few mutations were present with relatively higher frequencies. The N-terminal signal peptide region (the first 20 amino acids) and C-terminal cytoplasmic tail region were excluded for further analysis, as these either were cleaved during the protein maturation process or shielded intracellularly and, thus, functionally non-relevant to spike immunogenicity. Therefore, we eventually enlisted ancestral strains together with nine representative point mutations for a comprehensive analysis (Table 1).

### 3.2. Virological Features of Early Emerging Mutations in Spike Protein

**Figure 2:**
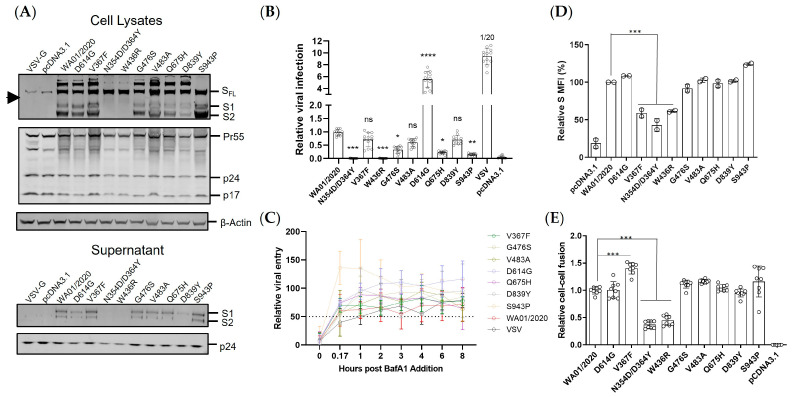
Virological features of early emerging mutations in spike protein. (**A**) The cell lysates from HEK293T cells transfected with psPAX2, pLenti-Luc, and spike plasmids were analyzed by Western blot (left). Meanwhile, the viral particles in supernatants were analyzed by Western blot (right). Internal control β-actin, HIV-1 p24, and coronavirus spike proteins were examined in both cell lysates and viral particles. Arrowhead indicates non-specific bands. (**B**) Lentiviral particles pseudotyped with SARS-CoV-2 spike, VSV-G, or empty (pcDNA3.1) were used to transduce HEK293T-hACE2 cells, the firefly luciferase activity was quantified 48 h post-infection. Data are represented as Mean ± SD of 12 replicates. Parameters in the early mutant groups were compared with the WA01/2020 group by one-way ANOVA tests. **** denotes *p* < 0.0001. (**C**) The inhibition curve of BafA1 against SARS-CoV-2 or VSV-G pseudovirus was generated by pre-incubating BafA1 for one hour and performing virus infection in HEK293T-hACE2 cells. Dotted lines interpolate 50% inhibition. The table on the right indicates the estimated time for 50% viral entry. (**D**) The same batch of cells was detached by 5 mM EDTA/DPBS digestion buffer and stained with anti-SARS-CoV-2 RBD antibody. The surface expression of spike proteins was analyzed by flow cytometry. The representative flow plot was analyzed by FlowJo software v9.9, and spike expression was quantified as geometric mean fluorescence intensity. Data are represented as Mean ± SD of 2 replicates. (**E**) Cell–cell fusion was determined by co-transfecting SARS-CoV-2 S, VSV-G, or empty (pcDNA3.1) with HIV-1 Tat-expressing plasmid into HEK293T cells; meanwhile, pQCXIP-hACE2 was transfected into TZM-bl cells. Cells were mixed in a 1:1 ratio, and the firefly luciferase activity was quantified 12 h post coculture. Data are represented as Mean ± SD of 6 replicates. Parameters in the early mutant groups were compared with the WA01/2020 group by one-way ANOVA tests. *** denotes *p* < 0.001.

The primary function of coronavirus spike protein is to mediate viral entry. To investigate whether this very early stage of SARS-CoV-2 infection was influenced by the emerging mutations, we exploited single-round lentiviral vector-based and spike protein pseudotyped reporter viruses. Notably, the last 13 amino acid residues in the cytoplasmic tail were removed to increase the spike incorporation into the pseudovirions [20]. The empty vector pcDNA3.1 was included as the negative control, while a VSV-G protein served as the virus-specific control. The protein expression profile was subsequently examined by Western blot in HEK293T cells transfected with backbone vector pLenti-Luc, packaging plasmid psPAX2, and spike constructs (Figure 2A). Despite that, the HIV gag proteins (Pr55 and p24) and loading control β-actin demonstrated a comparable expression in cell lysates. The spike proteins, determined by antibody cocktail recognizing both S1 and S2 regions, showed much heterogeneity, as reflected by the varied expression pattern of S1, S2, and full-length spike (SFL) bands (Figure 2A). Compared with WA01/2020, mutants such as D614G, V367F, G476S, V483A, Q675H, D839Y showed comparable expression pattern. S943P demonstrated an up-regulated total protein expression level, while oppositely mutants such as W436R and N354D/D364Y showed lower protein expression levels. Of particular interest, we assayed the ratio of S1 to SFL, a hallmark of the spike processing efficiency [28]. We noticed that W436R, N354D/D364Y, and, to a lesser extent, D839Y showed much diminished S1 and S2 expressions, while in contrast, the mutant S943P demonstrated enhanced S2/SFL ratios (Figure 2A). We further assayed the cell culture supernatants, which presumably contain the spike pseudovirions. We observed a generally comparable p24 secretion, indicating a similar level of lentiviral particle production. Interestingly, the three mutants with lower processing ratios in the cell lysates (W436R, N354D/D364Y, and D839Y) also showed fewer spike incorporations into the pseudovirions, while S943P showed slightly greater S1 and S2 participation (Figure 2A). Next, pseudoviruses were applied to HEK293T-ACE2 cells, and the viral entry was determined by firefly luciferase luminescence assay. Compared with ancestral WA01/2020, mutants, including V367F, V483A, Q675H, and D839Y, showed a viral entry efficiency of less than two-fold reduction (Figure 2B). Interestingly, three mutants, including G476S, Q675H, and S943P, showed greater than two-fold lower viral entry, while the viral entry of N354D/D364Y and W436R was severely impaired, with an efficiency close to mock control (Figure 2B). D614G is the only mutant that exhibited approximately six-fold enhanced viral entry, which aligns well with prior reports [23,29,30,31]. Given the heterogeneity of the mutants mediated viral entry, we sought to investigate their entry kinetics. By using a Bafilomycin A1 (BafA1) blocking assay, we observed that the coronavirus showed an accelerated entry process compared to VSV (Figure 2C and Table 2). Moreover, the early SARS-CoV-2 mutants demonstrated similar entry kinetics to WA01/2020, except that the S943P appeared to possess more rapid viral entry. In addition, the S943P reached the plateau more efficiently than other mutants (Figure 2C and Table 2).

Cell–cell fusion represents an essential means of viral transmission and a hallmark of viral pathogenesis [32]. We used a pre-established cell coculture-based assay to measure the fusogenicity of the spikes [20]. Given that the fusion events were initiated predominantly by ACE2-spike interaction on the plasma membrane, we first determined the spike expression by cell surface staining. In agreement with the whole protein expression pattern, the cell surface displayed spikes are comparable to WA01/2020 except that V367F, N354D/D364Y, and W436R showed a roughly two-fold reduction (Figure 2D and Figure S1). As expected, the comparable surface expression of spikes triggered comparable cell–cell fusion (Figure 2E), while the lower expression of spikes such as N354D/D364Y and W436R triggered less fusion formation. Interestingly, the V367F demonstrated elevated cell–cell fusion despite lower cell surface expression (Figure 2E).

**Figure 3:**
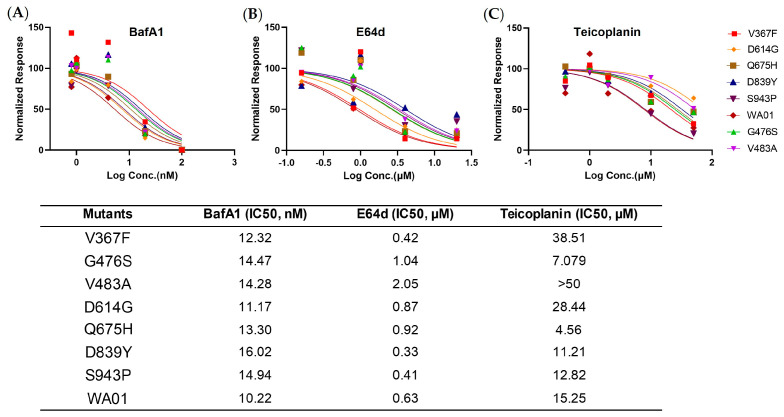
Sensitivity to early spike mutants to entry inhibitors. Inhibition curve of BafA1 (**A**), E64d (**B**), and Teicoplanin (**C**) against SARS-CoV-2 spike or VSV−G pseudovirus were generated by pre-incubating inhibitors for one hour and performing virus infection in HEK293T−hACE2 cells. Additionally, 50% inhibition concentration (IC50) was interpolated. Data are represented as Mean ± standard deviation of technical triplicates.

Finally, we assessed the sensitivity of these emerging mutants to a panel of coronavirus entry inhibitors, including the lysosomal inhibitor BafA1, Cathepsin L inhibitor teicoplanin, and Cathepsin B inhibitor E64d. Compared with the WA01/2020, the single-point mutants demonstrated equivalent IC50s to BafA1 (Figure 3A). Interestingly, V483A demonstrated enhanced IC50 to E64d (Figure 3B), while V367F showed elevated IC50 to Teicoplanin (Figure 3C). Q675H showed greater than two-fold enhanced sensitivity to Teicoplanin (4.56 μM vs. 15.25 μM) (Figure 3C), suggesting that these early mutants maintained similar viral endocytic entry pathways in the cell culture model but with mixed efficiency.

Taken together, the virological evidence demonstrated that these early mutants are divergent in protein expression and functioning while they kept conserved entry cascades.

### 3.3. Antibody Responses Elicited by Early Emerging Mutations in Spike Protein

**Figure 4:**
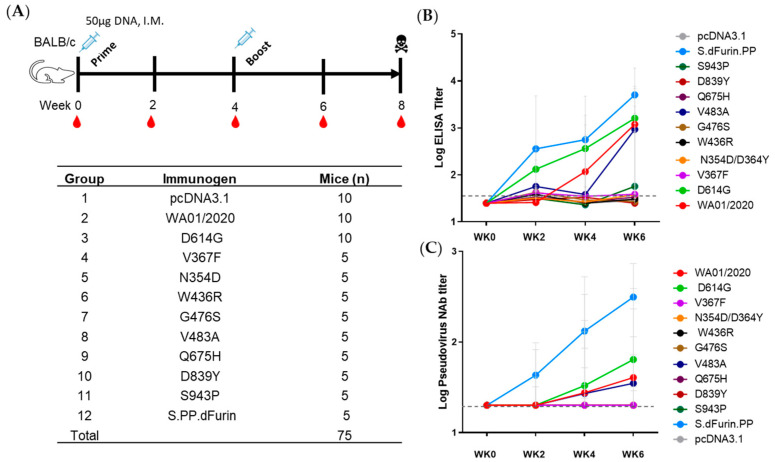
Immunogenicity of early emerging spike mutants. (**A**) Study schema. To explore the immunogenicity of these early mutants, wild-type BALB/c mice were immunized at week 0 and week 4 with 50 μg of plasmid DNA. Peripheral blood was collected at baseline and every two weeks following vaccination to monitor antibody responses in serum. Eight weeks post-prime, mice were sacrificed for cellular immune response determination. Vaccine groups and timing of immunization and challenge are shown in the table. (**B**) S-specific binding antibody responses were quantified through enzyme-linked immunosorbent assay (ELISA) in serum every two weeks post-prime. (**C**) The neutralizing activity of elicited antibody responses was assessed through pseudovirus SARS-CoV-2 neutralization assays. Individual mice are denoted by single dots, and the red bar reflects the median titer. Representative data from one of two similar experiments are shown. Parameters in the early mutant groups were compared with the WA01/2020 group by two-sided Mann–Whitney tests.

In addition to the viral entry, the spike is key to eliciting protective immunity in the host. To investigate whether early mutations would affect the immunogenicity of spikes, we intramuscularly immunized BALB/c mice with 50 μg DNA plasmids (Figure 4A). The mice were primed at week 0 and boosted at week 4. Bleeding was conducted at weeks 0, 2, 4, 6, and 8. At week 8, mice were sacrificed for terminal cellular immune response determination. Antibody induction was quantified by standard ELISA assay (Figure 4B) and pseudovirus-based neutralization assay (pNAb) (Figure 4C). S.dFurin.PP (S.PP), the conformation-stabilized version of spike proteins, was included as a positive control [33]. Consistent with our prior results, the S.PP demonstrated significantly sooner and greater antibody responses than unmodified versions (Figure 4B,C) [21,33]. For those unmodified, the binding antibody was detected at week 2 and the neutralizing antibody at week 4. Intriguingly, we noticed that only 3 out of 10 early mutants (WA01/2020, D614G, and, at a later time point, V483A) showed apparent antibody induction after the prime-boost regimen (Figure 4B,C). Moreover, the D614G mutant, which out-competed and replaced ancestral D614 as soon as its emergence, showed more rapid and pronounced antibody responses than the ancestral strain (Figure 4B,C). We were curious whether the single amino acid swap was readily discernible to the host and, thus, examined the capacity of two sets of serums elicited by WA01/2020 or D614G at week 8 in neutralizing reciprocal strains. As shown in Figure S2, although statistically non-significant, both the WA01/2020 serum and D614G serums exhibited a trend of greater neutralizing activity against homologous strains, suggesting that the host immune system may distinguish the single amino acid alteration. Collectively, these data indicate that most early emerging mutants are weak in eliciting antibody responses, but they can be strong, as in the case of D614G.

### 3.4. T Cell Immune Responses Elicited by Early Emerging Mutations in Spike Protein

**Figure 5:**
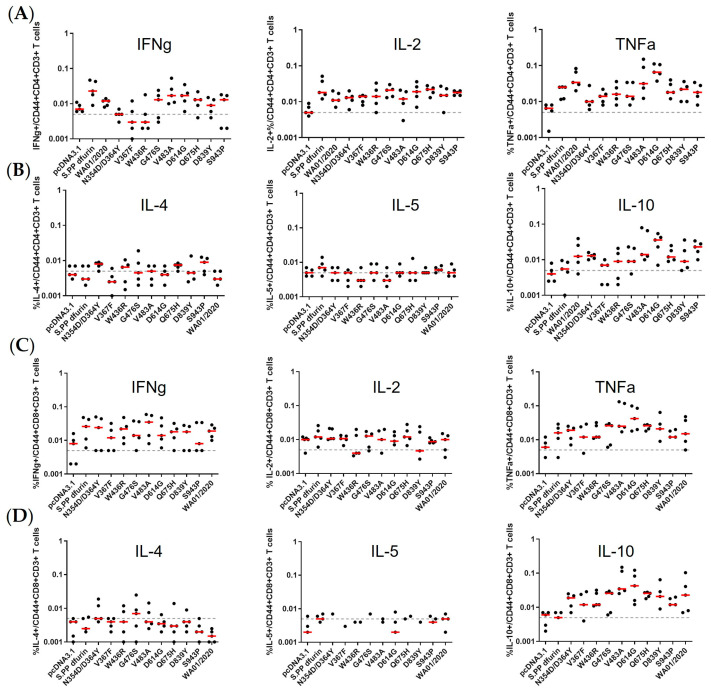
Cellular immune responses to early spike mutants. Spike-specific CD4+ T cell immune responses (**A**,**B**) and CD8+ T cell immune responses (**C**,**D**) were measured by intracellular staining in isolated spleen from BALB/c mice. IFNγ, IL-2, and TNF-α serve as the Th1 immune response marker (**A**,**C**), while IL-4, IL-5, and IL-10 serve as the Th2 immune response marker (**B**,**D**). Individual mice are denoted by single dots, and the red bar reflects the median titer. Representative data from one of two similar experiments are shown. Parameters in the early mutant groups were compared with the WA01/2020 group by two-sided Mann–Whitney tests.

We next determined the T cell immune responses by the DNA vaccination regimen. The spleen cells at week 8 were stained for both CD4 and CD8 T cell analyses by flow cytometry and ELISPOT assays. In CD4 subsets, generally comparable Th1 immune responses were observed, as reflected in the statistically non-significant induction of Th1-specific cytokines, IFNγ, IL-2, and TNFα (Figure 5A). The relative lower IFNγ and TNFα in N354D/D364Y, V367F, and W436R may reflect their compromised expression level in vivo. In addition, the Th2 immune responses in CD4 T subsets were quantified by IL-4, IL-5, and IL-10 secretion, which largely borderline the limit of detection (Figure 5B). A similar trend was able to extend to CD8 T cell immune responses. After peptide stimulation, we noticed similar secretion of Th1 and Th2 cytokines (Figure 5C,D). We also determined IFN-γ secretion after peptide stimulation by ELISPOT and found no significant difference among the early mutants. Taken together, the data suggest that the early emerging mutant spikes maintained the same dominant T cell epitopes as the ancestral strain and elicited comparable T cell immune responses.

### 3.5. Early Emerging Mutants Maintained the Same Sensitivity to Antisera and Type I IFN

**Figure 6:**
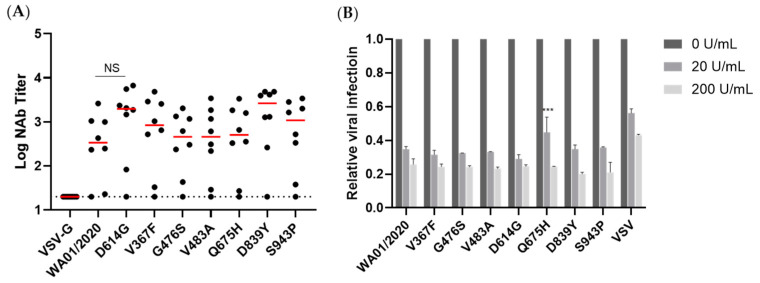
Sensitivity of the SARS-CoV-2 mutants to antisera and type I interferon. (**A**) A panel of serum from 8 mice immunized with SARS-CoV-2 protein nanoparticle vaccines was measured for neutralizing activity against early spike mutants. Data demonstrated a 50% neutralization titer, and the red bar reflects the median titer. Parameters in the early mutant groups were compared with the WA01/2020 group by two-sided Mann–Whitney tests. NS, not statistically significant. (**B**) 0, 20, and 200 U/mL IFNα2b were applied to the HEK293T-ACE2 cells for 12 h, and the pseudovirus-based viral entry was determined. The viral entry of each mutant with mock treatment was set as 1. *** denotes *p* < 0.001.

Immune evasion remains the top concern regarding the SARS-CoV-2 mutants. We are curious whether these early mutants that emerged before the vaccine deployment or reinfection would show resistance to antisera from a panel of ancestral spike nanoparticle immunized mice. As shown in Figure 6A, the neutralizing activity against early emerging mutants was not statistically different, implicating that the mutants may not have been subject to immune selection. In addition, we examined the sensitivity of lenti-viral-based pseudovirus to interferon α2b (IFNα2b), one of the key innate immune anti-viral players. Interestingly, we noted that the VSV showed relatively greater resistance to IFNα2b compared with the SARS-CoV-2 spikes. Moreover, the single mutants generally demonstrated a comparable sensitivity to IFNα2b, except that Q675H showed elevated resistance to 20 U/mL IFNα2b (Figure 6B). Together, the data here implicated that the early emerging mutants likely maintained the same sensitivity to both innate immunity and adaptive immunity.

## 4. Discussion

The genomic surveillance of SARS-CoV-2 is crucial for the timely detection and characterization of circulating variants, as well as monitoring the emergence of new strains [34]. Moreover, functional validation of the emerging and accumulating mutations can be equally important. In this study, we snapshot spike substitutions that occurred in the first four months of the pandemic and analyzed the virologic and immunologic characteristics of nine representative mutants, revealing that the virologic features are rather diversified while the immunologic traits are largely maintained.

The spike expression level and the viral entry efficiency are two determinants of viral fitness. Under the presumable low immune pressure, the early mutants demonstrated surprisingly virologic heterogeneity. First, the protein expression either in total or on the cell surface varied even with only a single amino acid substitution, with 1 out of 9 (11%) up-regulated, 2 out of 9 (22%) down-regulated, and 6 out of 9 (67%) neutral. The mutations in the spike were blueprinted on the spike from the ancestral strain WA01/2020, which were codon optimized to allow optimal expression in human cells. Therefore, this heterogenous expression pattern of spike mutants may reflect the protein folding constraints and/or accelerated turnover instead of translation blockage [35]. Second, the early mutants also showed an altered S1 to SFL ratio, implicating potentially compromised spike processing in the ER or Golgi apparatus. Third, functional characterization of viral entry by a lentiviral vectored pseudovirus demonstrated that the majority of the mutants mediated comparable viral entry to the ancestral spike, while the mutants N354D/D364Y and W436R manifested severe reduction, likely due to the compromised protein expression and/or processing. It remains to be determined how the N354D/D364Y and W436R substitution influenced the spike expression/processing. It could be due to a change of structural conformation that leads to altered accessibility to the furin cleavage site, the perturbed glycosylation that leads to reduced protein thermostability [36], or other reasons. Conversely, the S943P showed greater S2 expression but lower infectivity. We reasoned that the S943P mutation likely leads to a conformation change on the spike that disfavors the pre-fusion process of viral entry, which warrants further validation studies. Interestingly, prior reports demonstrated that the V367F RBD showed elevated expression compared with the ancestral strain [35]. However, we observed a much-diminished pattern. The full-length spike likely confers more constraints than the smaller RBD during the protein folding process. If that is the case, the previously built model for protein expression prediction may need to be optimized based on a full-length spike instead of RBD. We also noted that the well-characterized D614G showed much elevated viral entry than the parental and mutants, which may help to explain its rapid replacement of the ancestral strain and was considered the key mutation that makes the virus more transmissible worldwide. Cell–cell fusion represents an essential means of virus transmission, particularly in in vivo settings [32]. The mutants N354D/D364Y and W436R again showed lower fusogenicity than the other mutants, likely resulting from a lower spike presence on the plasma membrane (Figure 2C). Interestingly, the D614G showed comparable fusion to the WA01/2020 despite an approximate six-fold elevated viral infectivity. Moreover, the V367F, even though it showed lower protein expression and viral entry, induced 50% greater cell–cell fusion. Therefore, not only the spike protein itself but also the microenvironment where it resides can significantly impact its biological functioning.

The immunogenicity of spikes was assessed via a prime-boost DNA immunization regimen. We observed only WA01/2020, D614G, and, to a lesser extent, V483A elicited binding and neutralizing antibody responses, leaving other mutants inert at least 6 weeks post-prime immunization. The mutants N354D/D364Y and W436R may be less immunogenic since their expression level are lower (Figure 2A). However, these suppressed antibody responses by mutants with steady or elevated expression are unexpected. The apparent induction of T cell immune responses argues against the possibility of compromised in vivo expression as well (Figure 5). Whether this reflects a genuine difference between in vivo and in vitro expressions or is confined to a murine model warrants future characterization. This observation, on the other hand, may implicate that the early mutants may shield themselves from recognition by the host immune system and, thus, emerge. In addition, we take particular interest in the D614G mutation since it entirely replaced the ancestral strains shortly after its emergence [23]. We noted that D614G induced more rapid and pronounced antibody titers than the ancestral strain. In addition, this single amino acid change is enough for the host immune system to differentiate. This piece of data suggests that this single amino acid change may cause a change of conformation [31,37], leading to favorable T and B cell activation. Regarding T cell responses, we noted generally comparable CD4+ T and CD8+ T cell responses, supporting recent reports that T cell epitopes are relatively conserved [6,38].

To gain a more comprehensive picture of the spike, we determined the viral entry kinetics and its sensitivity to viral inhibitors that block ACE2, Cathepsin B, or Cathepsin L, respectively. In general, most mutants tested demonstrated a similar half-maximal inhibitory concentration (IC50) and entry half-life to the ancestral WA01/2020, indicating limited selection pressure on these early mutants. In line with the virologic data, a panel of antisera from spike protein-immunized mice showed comparable neutralizing activity against all mutants, implicating a low chance of immune evasion. Similarly, the mutants showed a comparable type I IFN sensitivity. Therefore, these early mutants likely emerged sporadically and remain sensitive to antivirals and host immunity.

Our study has several limitations. First, our comparative fitness analysis was based on the sequence data released in GISAID and is, thus, subject to the selection bias of sequences being released to the public database. The proportion of SARS-CoV2 sequences varied substantially by sampling time and location, and therefore, we limited our analysis to the circulating period of the strains before 15 March 2020. In addition, the information based on spike mutation may not apply to authentic infectious viruses, since the mutations on other viral genes may deeply impact the viral fitness and shape the overall landscape collectively with spike mutations. Finally, all mutants except D614G were not present in the subsequent VOCs, again indicating limited immune pressure at the time of sampling. However, the early emerging mutations do formulate a pool, which supplied a more rapidly spreading infection, such as D614G and V367F in the case of viral infection and cell–cell fusion, respectively. Thus far, it would be necessary to keep surveilling the early emerging mutants and their viral fitness potential in future pandemics.

Together we snapshot a few mutants that emerged at the early phase of the pandemic and observed intriguingly divergent protein expression, viral entry, cell–cell fusion activity, and immunogenicity. This information can be useful in guiding vaccine design and future pandemic preparedness.

## Figures and Tables

**Table 1:** Features of early spike mutants.

Mutants	Frequency	Region	Existing Report
D614G	+++++	S1 C-terminus	Enhanced viral entry [23]
V483A	+++	RBD	Enhanced binding to receptor [24] Antibody resistance [25]
S943P	+++	HR1	NA
D839Y	+++	Fusion peptide	Antibody resistance [26]
G476S	++	RBD	Reduced affinity to receptor [15]
Q675H	++	S1 C-terminus	Enhanced viral entry [27]
V367F	++	RBD	Enhanced receptor binding [13]
W436R	+	RBD	Enhanced receptor binding [14]
N354D/D364Y	+	RBD	Enhanced receptor binding [14]

**Table 2:** Estimates of time of 50% viral entry.

Mutants	Time of 50% Viral Entry (min)
V367F	<10
G476S	~10
V483A	~10
D614G	<10
Q675H	<10
D839Y	~10
S943P	<5
WA01	~10
VSV	60

## Data Availability

All data are available in the manuscript or the Supplementary Materials. Correspondence and requests for materials should be addressed to J.Y. (yu_jingyou@gzlab.ac.cn).

## Supplementary Materials

The following supporting information can be downloaded at: https://www.mdpi.com/article/10.3390/v15122401/s1, Figure S1: Spike cell surface expression; Figure S2: the reciprocal neutralizing activity of antisera against WA01/2020 or D614G was determined.
