# Supplementary material for: Early Emerging SARS-CoV-2 Spike Mutants Are Diversified in Virologic Properties but Elicit Compromised Antibody Responses
Source: Viruses. 2023 Dec 9;15(12):2401. doi: 10.3390/v15122401 (PMC10747620; doi:10.3390/v15122401)
Supplement: Supplementary file 1 [file viruses-15-02401-s001.zip › viruses-2758258-supplementary.pdf]

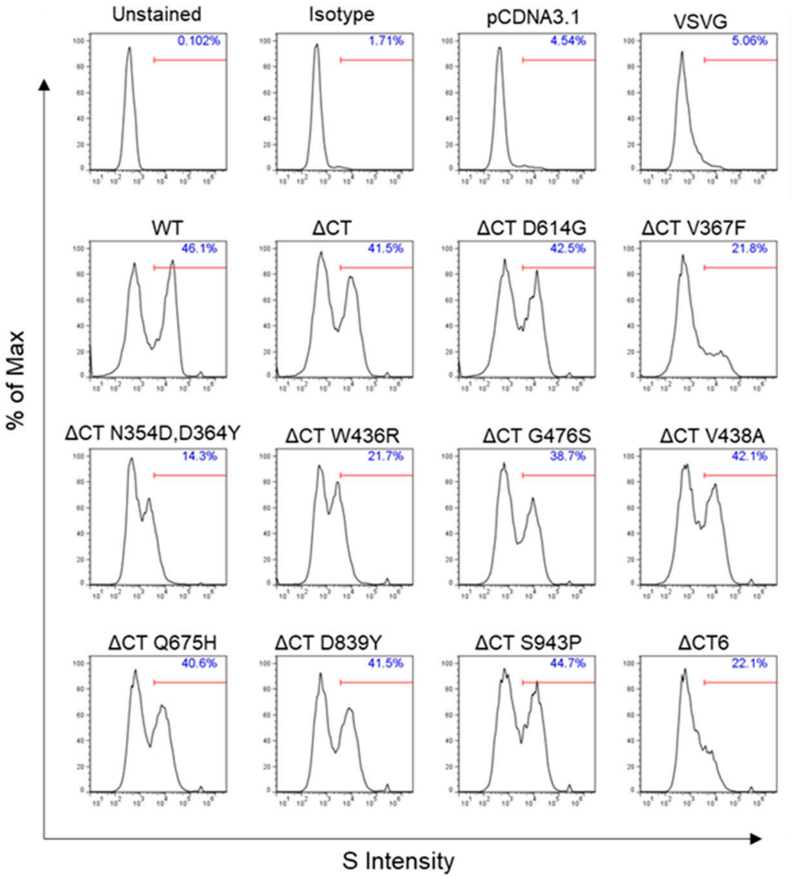

Figure S1. Spike cell surface expression. HEK293T cells transfected with spike plasmids was detached by 5 mM EDTA/DPBS digestion buffer and stained with an-ti-SARS-CoV-2 RBD antibody. The surface expression of spike proteins was analyzed by flow cytometry. The representative flow plot was analyzed by FlowJo and gating by spike fluroscent intensity.

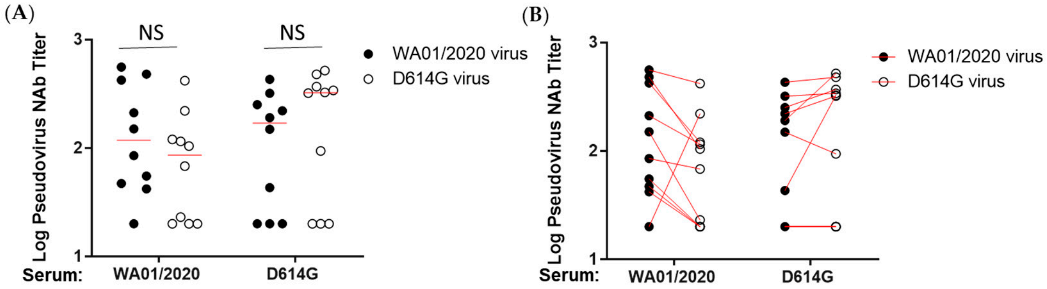

Figure S2. The reciprocal neutralizing activity of antisera against WA01/2020 or D614G was determined. Red lines indicate the median titer of each group. Parameters in the early mutant groups were compared with the WA01/2020 group by two-sided Mann-Whitney tests. NS, not statistically significant.
